# Development of a tag-free plant-made interferon gamma production system with improved therapeutic efficacy against viruses

**DOI:** 10.3389/fbioe.2023.1341340

**Published:** 2024-01-11

**Authors:** Min-Chao Jiang, Wei-Li Hsu, Ching-Yu Tseng, Na-Sheng Lin, Yau-Heiu Hsu, Chung-Chi Hu

**Affiliations:** ^1^ PhD Program in Microbial Genomics, National Chung Hsing University and Academia Sinica, Taichung, Taiwan; ^2^ Graduate Institute of Microbiology and Public Health, College of Veterinary Medicine, National Chung Hsing University, Taichung, Taiwan; ^3^ Institute of Plant and Microbial Biology, Academia Sinica, Taipei, Taiwan; ^4^ Graduate Institute of Biotechnology, National Chung Hsing University, Taichung, Taiwan; ^5^ Advanced Plant Biotechnology Center, National Chung Hsing University, Taichung, Taiwan

**Keywords:** viral vector, therapeutic protein, *Bamboo mosaic virus* (BaMV), cleavable peptide, tag-free IFNγ, antivirus activity

## Abstract

Plants offer a promising platform for cost-effective production of biologically active therapeutic glycoproteins. In previous studies, we have developed a plant expression system based on *Bamboo mosaic virus* (BaMV) by incorporating secretory signals and an affinity tag, which resulted in notably enhanced yields of soluble and secreted fusion glycoproteins (FGs) in *Nicotiana benthamiana*. However, the presence of fusion tags on recombinant glycoproteins is undesirable for biomedical applications. This study aimed to develop a refined expression system that can efficiently produce tag-free glycoproteins in plants, with enhanced efficacy of mature interferon gamma (mIFNγ) against viruses. To accommodate the specific requirement of different target proteins, three enzymatically or chemically cleavable linkers were provided in this renovated BaMV-based expression system. We demonstrated that *Tobacco etch virus* (TEV) protease could process the specific cleavage site (L_TEV_) of the fusion protein, designated as SS^Ext^His(SP)_10_L_TEV_-mIFNγ, with optimal efficiency under biocompatible conditions to generate tag-free mIFNγ glycoproteins. The TEV protease and secretory-affinity tag could be effectively removed from the target mIFNγ glycoproteins through Ni^2+^-NTA chromatography. In addition, the result of an antiviral assay showed that the tag-free mIFNγ glycoproteins exhibited enhanced biological properties against *Sindbis viru*s, with comparable antiviral activity of the commercialized HEK293-expressed hIFNγ. Thus, the improved BaMV-based expression system developed in this study may provide an alternative strategy for producing tag-free therapeutic glycoproteins intended for biomedical applications.

## 1 Introduction

Harnessing recombinant proteins for biomedical applications is among the central goals of molecular bioengineering. To achieve this, scientists have developed diverse expression systems, using organisms like animals, yeasts, and bacteria, to efficiently produce a range of therapeutic proteins (Berlec and Strukelj, 2013; Wells and Robinson, 2017). Plants may serve as alternative biofactories for producing therapeutic proteins due to their unique advantages in high safety, rapid scaling-up, and cost-efficient production (Daniell et al., 2001; Schillberg et al., 2019). In addition, plant-made pharmaceuticals (PMPs) allow for eukaryotic post-translational modification (PTM), which is essential for protein stability, maturation, and biological function (Walsh and Jefferis, 2006; Schneider et al., 2014; Liu and Timko, 2022). However, one major drawback associated with PMP production is the lack of efficient downstream purification processes for achieving biomedical acceptance.

To overcome challenges in downstream processing of plant-produced recombinant proteins, the use of various fusion tags has emerged as a valuable strategy. For example, a small ubiquitin-related modifier (bdSUMO) derived from *Brachypodium distachyon* (Islam et al., 2019; Islam et al., 2020), an elastin-like polypeptide (ELP) (Conley et al., 2009; Kaldis et al., 2013; Marin Viegas et al., 2022), and hydrophobins (HFBI) derived from *Trichoderma reesei* (Joensuu et al., 2010; Reuter et al., 2014) have been used to significantly improve productivity in plant-based systems. The combination of secretory signal (SS) and a plant-specific glycomodule tag, hydroxyproline (Hyp)-O-glycosylated peptide (HypGP) consisting of a repeated “Ser-Pro” motif, has resulted in a 16-fold increase in the overall yield of the tagged protein when transiently expressed in inoculated *N. benthamiana* leaves (Dolan et al., 2014). Such tags have been used for boosting secretion, which could dramatically promote the production of secreted recombinant proteins in various plant cell culture systems, including tobacco BY2 suspension cells (Xu et al., 2007; Xu et al., 2010; Zhang et al., 2016), green microalga *Chlamydomonas reinhardtii* (Ramos-Martinez et al., 2017), and tobacco hairy root cultures (Zhang et al., 2019). In particular, the use of secretory plant cell culture systems is applicable in large-scale and continuous production of therapeutic glycoproteins in compliance with the current good manufacturing practice (CGMP) regulations.

In our previous study, by using an overexpression system based on *Bamboo mosaic virus* (BaMV), we incorporated a novel secretory signal, SS^Ext^, derived from *N. benthamiana* extensin protein (Jiang et al., 2020) to transport the mature interferon gamma (mIFNγ) protein via the secretory pathway for maintaining intact post-translational modification. Additional fusion of the HypGP tag, (SP)_10_, which contains 10 repeats of the “Ser-Pro” motif of hydroxyproline-O-glycosylated peptides, to the C-terminus of mIFNγ dramatically enhanced the solubility and secretion of the fusion glycoprotein (FG), designated as SS^Ext^mIFNγ(SP)_10_. This strategy has simplified the downstream purification process for scaled-up production of FG in *N. benthamiana* plants (Jiang et al., 2020). It was noted that the fusion protein SS^Ext^mIFNγ(SP)_10_ was partially O- or N-glycosylated and post-translationally modified to various forms such as monomeric mIFNγ (Mγ, 16 kDa), monomeric monoglycosylated mIFNγ (1N-MG, 18 kDa), and monomeric diglycosylated mIFNγ (2N-MG, 20 kDa), which then underwent self-assembly into the dimeric glycosylated mIFNγ (DG, 32–40 kDa). However, the fusion tags on SS^Ext^mIFNγ(SP)_10_ proteins were still not completely processed through the secretory pathway, as confirmed by immunoblotting with specific antibodies. These FG forms would possibly affect their biological activity due to their undesirable protein folding conformations.

One solution to increase the efficiency in the processing of fusion proteins (FPs) is through the insertion of cleavable peptides in between fusion tags and recombinant proteins, such as the motifs recognized by *Staphylococcus aureus* sortase A (SrtA) (Ton-That et al., 1999; Tsukiji and Nagamune, 2009), *Tobacco etch virus* (TEV) protease, enterokinase, or factor Xa (Arnau et al., 2006; Waugh, 2011). TEV protease was widely applied in the removal of fusion tags from FPs since it exhibits high enzymatic activity even at low temperature (4°C) (Arnau et al., 2006). Due to steric occlusion between the fusion partner and target protein, some FPs, particularly membranous ones, could not be cleaved by proteases even in the presence of the specific cleavage site (Waugh, 2011). For such membranous FPs, a new sequence-specific nickel-assisted cleavage (SNAC) tag has been developed as a chemically cleavable linker in FPs, which could efficiently remove the fusion partner by the Ni^2+^ ion under biocompatible conditions (Dang et al., 2019). Apparently, different target proteins may pose different challenges for processing, and there is still need for development of alternative strategies to process the fusion tags to accommodate the different requirements of diverse recombinant proteins.

In this work, we intended to develop a BaMV-based expression system with different cleavable fusion tags to suit the specific needs of target proteins and tested the applicability of the system for obtaining tag-free mIFNγ glycoproteins suitable for biomedical usages. We introduced three different enzymatically or chemically cleavable linkers mentioned above, designated as L_SrtA_, L_TEV_, and L_SNAC_, in between the combinational secretory-affinity tag (SS^Ext^His(SP)_10_) and mIFNγ in the BaMV-based overexpression system. The results revealed that the fusion glycoprotein, designated as SS^Ext^His(SP)_10_L_TEV_-mIFNγ, expressed by the BaMV-based vector in *N. benthamiana,* was successfully produced, and the tags were proteolytically cleaved by TEV protease with optimal efficiency. A series of purification procedures were established for the efficient production of tag-free mIFNγ glycoproteins, which were further verified to exhibit improved biological activity against *Sindbis viru*s.

## 2 Materials and methods

### 2.1 Construction of BaMV-based expression cassettes for FG processing

**FIGURE 1:**
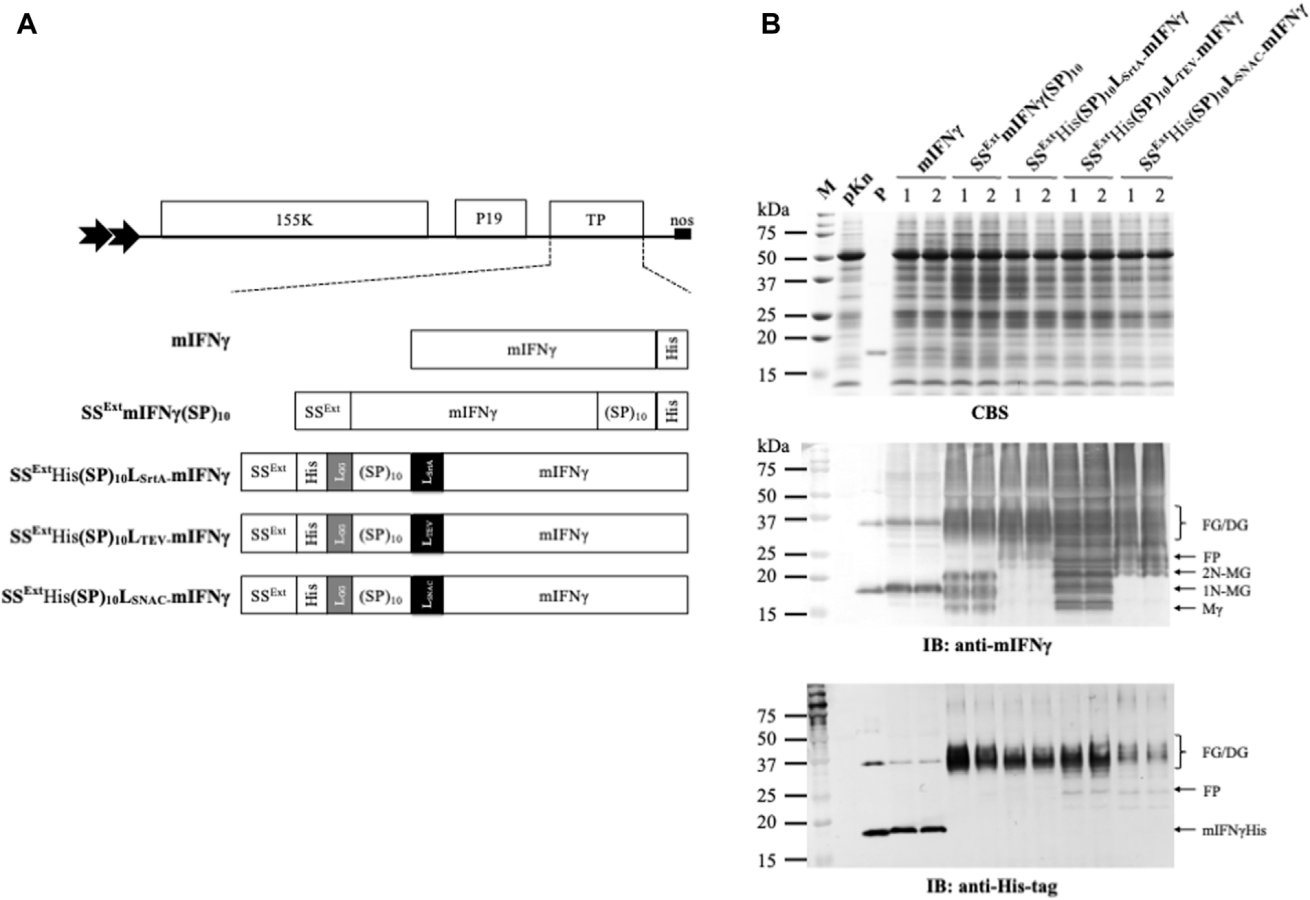
Effect of different cleavable linkers on the transient expression of various fusion Glyco-mIFNγ (FGs) in *N. benthamiana*. **(A)** Schematic representation of the renovated BaMV-based secretory expression cassettes, with the cleavable linker (L_SrtA_, L_TEV_, or L_SNAC_) inserted between the combinational secretory-affinity tag (SS^Ext^His(SP)_10_) and mIFNγ. A short peptide, LGG (denoted by the gray boxes) (Chen et al., 2013), was used as a linker between SS^Ext^-His and (SP)_10_. The transcription of the BaMV-based vector supplemented with the RNA silencing suppressor, P19, was under the control of a dual 35S promoter from the *Cauliflower mosaic virus* (denoted by the double arrowheads) and a nopaline synthase (nos) terminator. **(B)** Analysis of FG expression in infiltrated leaves at 5 days post-infiltration (5 dpi). Total proteins were extracted from infiltrated *N. benthamiana* leaves, separated by electrophoresis through a 12% polyacrylamide gel containing 1% SDS (SDS-PAGE), followed by visualization with Coomassie blue staining (CBS) and immunoblotting (IB) analysis with alkaline phosphatase (AP)-conjugated antibodies specific to mIFNγ or His-tag. M, marker; pKn, vector only; P, purified mIFNγ protein derived from *E. coli* as a positive control; FG, SS^Ext^His(SP)_10_L-mIFNγ glycoprotein; FP, SS^Ext^His(SP)_10_L-mIFNγ protein; Mγ, monomeric mIFNγ; 1N-MG, M monoglycosylated mIFNγ; 2N-MG, M diglycosylated mIFNγ; DG, dimeric mIFNγ glycoprotein.

The improved BaMV-based expression vector developed in this study was modified from a previously constructed chimeric BaMV vector, pKB19 (Muthamilselvan et al., 2016; Jiang et al., 2019), which harbors BaMV RNA replicase, the silencing suppressor P19 of *Tomato bushy stunt virus* (TBSV) (519 bp, GenBank Accession No. AJ288926) under the control of a dual constitutive 35S promoter of *Cauliflower mosaic virus* (CaMV), and an *Agrobacterium* nopaline synthase (nos) terminator (Figure 1A). In addition, the SS^Ext^ derived from the *N. benthamiana* extensin protein with the fusion of the secretory booster, (SP)_10_, was incorporated into the expression vector to generate the secretory expression vector, pKB19SS^Ext^His(SP)_10_, which was further used to construct the plasmid pKB19SS^Ext^His(SP)_10-_mIFNγ for the production of human mIFNγ glycoproteins (amino acid positions 21–166, GenBank accession number AY121833.1) with enhanced solubility and secretion (Jiang et al., 2020). However, such fusion tags may affect the conformation and biological activity of the recombinant mIFNγ glycoproteins by hampering the proper folding. Thus, in this study, we have improved the design of the BaMV-based protein expression system by the insertion of cleavable linkers, including L_SrtA_, L_TEV_, and L_SNAC_, positioned in between the combinational secretory-affinity tag SS^Ext^ His(SP)_10_ [containing a 6X His-tag between SS^Ext^ and (SP)_10_] and mIFNγ to generate plasmids designated as pKB19SS^Ext^His(SP)_10_L_SrtA-_mIFNγ, pKB19SS^Ext^His(SP)_10_L_TEV-_mIFNγ, and pKB19SS^Ext^His(SP)_10_L_SNAC-_mIFNγ, respectively (Figure 1A). The construction process of the above plasmids is described in brief as follows. The fragment of 6X His-tag and (SP)_10_L_SrtA_-mIFNγ were synthesized by overlapping PCR with specific primers, including F-*Xba*I-LPETG-mIFNγ, F-(SP)_10_-*Xba*I-LPETG, F-*Mlu*I-(SP)_10_, and F-*Mlu*I-6XHis-GG-(SP)_10_, individually plus R-SpeI-TGA-mIFNγ (Supplementary Table S1). The amplified PCR fragment of His(SP)_10_L_SrtA_-mIFNγ, containing 6X His-tag at the N-terminus, was digested with *Mlu*I and *Spe*I and ligated into the respective sites in the pKB19 expression vector to generate the chimeric plasmid pKB19His(SP)_10_L_SrtA_-mIFNγ. The SS^Ext^ signal was constructed by primer extension of two mutually complementary primers, F-*Mlu*I-SS^Ext^ and R-*Mlu*I-SS^Ext^, followed by digestion with *Mlu*I. The digested product of the SS^Ext^ fragment was cloned into pKB19His(SP)_10_L_SrtA_-mIFNγ to generate the final chimeric plasmid pKB19SS^Ext^His(SP)_10_L_SrtA_-mIFNγ. The fragments of L_TEV_-mIFNγ and L_SNAC_-mIFNγ were amplified with the mIFNγ gene as a template using gene-specific forward primers, namely, F-*Xba*I-ENLYFQG-mIFNγ and F-*Xba*I-GSHHW-mIFNγ, respectively, plus the reverse primer R-*Spe*I-TGA-mIFNγ (Supplementary Table S1). The amplified PCR product fragments of L_TEV_-mIFNγ and L_SNAC_-mIFNγ were digested with *Xba*I and *Spe*I and ligated into the pKB19SS^Ext^His(SP)_10_ secretory expression vector cut with cognate enzymes to generate the chimeric plasmids pKB19SS^Ext^His(SP)_10_L_TEV_-mIFNγ and pKB19SS^Ext^ His(SP)_10_L_SNAC_-mIFNγ, respectively.

### 2.2 Transient expression of FGs in *N. benthamiana* plants

The transient expression of recombinant proteins in *N. benthamiana* was achieved using *Agrobacterium*-mediated infiltration. *A. tumefaciens* (PGV3850 strain) harboring the expression constructs of different FGs were cultured, harvested by centrifugation at 12,000 rpm for 1 min, and recovered in agro-infiltration buffer (10 mM MES and 10 mM MgCl_2_, pH 5.5) to reach appropriate OD_600_ for each construct. The culture solutions were then infiltrated into 6-week-old *N. benthamiana* plants by a syringe- or vacuum-infiltration process (Jiang et al., 2020). The infiltrated plants were individually maintained in the culture room at 28°C for 16-h light/8-h dark intervals.

### 2.3 Immunoblotting analysis

The infiltrated leaves were harvested at 5 days post-infiltration (dpi), and the total proteins extracted with 1:2.5 (w/v) protein extraction buffer (50 mM Tris-HCl, pH 8.0, 10 mM MgCl_2_, 10 mM KCl, 1 mM EDTA, 20% glycerol, 10% β-mercaptoethanol, and 2% SDS). The extracted total proteins were analyzed by electrophoresis through a 12% polyacrylamide gel containing 1% sodium dodecyl sulfate (SDS-PAGE), followed by visualization using Coomassie blue staining (CBS) and analysis by immunoblotting (IB). The rabbit primary antibodies against His-tag antibodies (1:5000 dilution) or mIFNγ (1:5000 dilution) were used in IB for the determination of FGs or mIFNγ glycoprotein accumulations, as described previously (Jiang et al., 2019).

### 2.4 Separation of soluble FGs from *N. benthamiana* leaf homogenates

For isolation of the soluble FGs (SS^Ext^His(SP)_10_L_SrtA_-mIFNγ or SS^Ext^His(SP)_10_L_TEV_-mIFNγ), the infiltrated leaves were harvested at 5 dpi. Leaf homogenates were prepared with the use of 1:2 (w/v) extraction buffer A [50 mM Tris–HCl, pH 7.6, 120 mM KCl, 15 mM MgCl_2_, 0.1 mM PMSF, 20% glycerol, 0.1% β-mercaptoethanol, and one tablet of cOmplete™ EDTA-free proteinase inhibitor cocktail (Roche Life Science, Penzberg, Germany)] (Osman and Buck, 1996). The homogenized extracts were filtered through a layer of Miracloth and centrifuged at 30,000 × g for 30 min to separate the pellet (designated P30) and supernatant (designated S30) fractions. To remove the abundant RuBisCO protein contaminations, the S30 fraction was treated with ultrapure acetic acids to achieve a pH value of 5.1 and then subjected to centrifugation at 30,000 × g for 30 min to obtain the P30-treated (P30t) and S30-treated (S30t) fractions as described previously (Park et al., 2015; Jiang et al., 2020). After the removal of most RuBisCO protein contaminations, the pH of soluble FGs in the S30t fraction was adjusted to 7.0 by adding 1 M NaOH to avoid protein degradation.

### 2.5 Preliminary purification of soluble FGs in a Ni^2+^-NTA column

To purify the FGs (SS^Ext^His(SP)_10_L_SrtA_-mIFNγ or SS^Ext^His(SP)_10_L_TEV_-mIFNγ glycoproteins) from the S30t fraction, the soluble FGs were vacuum-filtered through a 0.22-μm Millipore membrane and applied to a Ni^2+^-NTA column according to the manufacturer’s instructions. All fractions were collected and brought to equal volume with protein sample buffer (with 2% SDS) and assayed by SDS-PAGE, followed by visualization with CBS and IB with specific antiserum. The fractions containing the target FGs were pooled and subjected to further processing.

### 2.6 Proteolytic cleavage of SS^Ext^His(SP)_10_L_SrtA_-mIFNγ using sortase A

For the removal of the L_SrtA_-linked secretory-affinity tag (SS^Ext^His(SP)_10_) from the target protein, 1 μg of FG [SS^Ext^His(SP)_10_L_SrtA_-mIFNγ] was treated with SrtA, produced in the laboratory from *E. coli* harboring the SrtA overexpressing plasmid purchased from Addgene (Watertown, MA, United States). The multifunctional SrtA exhibits cysteine protease, protein ligase, and transpeptidase activities, specifically recognizing the LPET/G motif (the “/” sign denotes the cleavage site) in proteins (Ton-That et al., 1999; Tsukiji and Nagamune, 2009). To determine the optimal reaction condition, two-fold diluted SrtA (from 4 to 0.25 μg/μL) was incubated with 1 μg of the purified SS^Ext^His(SP)_10_L_SrtA_-mIFNγ in reaction buffer (50 mM Tris-HCl, pH 8.0, 150 mM NaCl, 10 mM CaCl_2_, and 4 mM β-mercaptoethanol) at 28°C overnight. The processed proteins were subjected to further purification as described below.

### 2.7 Proteolytic cleavage of SS^Ext^ (SP)_10_L_TEV_-mIFNγ using TEV protease

To remove the L_TEV_-linked tag, 10 mg of SS^Ext^His(SP)_10_L_TEV_-mIFNγ was treated with 0.2 mg of TEV protease as described previously (Kapust and Waugh, 1999). The TEV protease exhibits high cysteine protease activity under a wide range of reaction conditions (pH, salt, and temperature), which recognizes the specific ENLUFQG/S motif. The TEV protease concentrations were diluted either two-fold from 4 to 0.25 μg/μL or five-fold from 1 to 0.008 μg/μL to determine the optimal reaction condition. Each diluted TEV protease was added to 1 μg of the purified SS^Ext^His(SP)_10_L_TEV_-mIFNγ in reaction buffer (50 mM Tris-HCl, pH 8.0, 5 mM DTT, and 0.5 mM EDTA) at 4°C overnight. The processed proteins were subjected to further purification to extract the target protein.

### 2.8 Further purification of target FGs by chromatography through a second Ni^2+^-NTA and Superdex™ 200 pg (S200) column

The above protease-treated FGs in the reaction mixture were passed through a second Ni^2+^-NTA agarose column for three times, and the target mIFNγ glycoproteins were collected in the unbound fractions (flow-through). The Ni^2+^-NTA agarose was washed once with 10 mL washing buffer (20 mM 50 mM Tris–HCl, pH 8.0, 150 mM NaCl, and 20 mM imidazole), and the 6x His-tagged proteases (sortase A or TEV protease) bound to the Ni^2+^-NTA agarose were subsequently eluted for recycling with 10 mL of elution buffer A (20 mM 50 mM Tris–HCl, pH 8.0, 150 mM NaCl, and 100 mM imidazole) and 10 mL of elution buffer B (20 mM 50 mM Tris–HCl, pH 8.0, 150 mM NaCl, and 300 mM imidazole). The flow-through fractions containing tag-free mIFNγ glycoproteins were collected and then loaded on a HiLoad™ 16/60 Superdex™ 200 pg (S200) using an FPLC system (AKTA Purifier, GE Healthcare, IL, United States) in S200 buffer (50 mM Tris-HCl, pH 8.0, 200 mM NaCl, and 5 mM β-mercaptoethanol) at a flow rate of 0.5–1 mL/min. The FG-containing fractions were pooled and further concentrated using a 10-kDa NMWL Centricon filter (GE Healthcare, IL, US). The concentrated mIFNγ glycoproteins were dialyzed with PBS buffer for further biological activity assay.

### 2.9 Protein quantification

The quantification of all purified proteins was confirmed by using different methods, as followed by a previous study (Jiang et al., 2019; Jiang et al., 2020), including Bradford colorimetric assay, ELISA, and Coomassie blue staining (CBS) following SDS-PAGE.

### 2.10 Infection of *Sindbis virus* in cells treated with interferon–gamma (IFN-γ) variants

To investigate the biological activity of recombinant IFN-γ variants, an anti-SINV assay was performed, as described previously (Jiang et al., 2020), using the reporter *Sindbis virus* carrying an enhanced green fluorescent protein expression cassette (designated as SINV-eGFP) kindly provided by Professor Lih-Hwa Hwang (Graduate Institute of Microbiology and Immunology, National Yang-Ming University, Taipei, Taiwan). In brief, HEK293 cells (2.5 × 10^5^) were seeded in a 24-well plate 8 h prior to treatment. The cells were washed twice with PBS and treated with DMEM (Mock), the extract from healthy leaves of *N. benthamiana* as the negative control (NC), or five-fold serial dilutions (from 50 ng/mL to 0.08 ng/mL) of commercial mIFN-γ (Accession No. CAA31639) produced from *E. coli* as a positive control (PC-IFN-γ), another PC-IFN-γ (Accession No. NP_000610.2) produced from HEK293 (R&D Systems, Inc., MN, United States), or three IFN-γ variants (SSmIFNγ(SP)_10_, SSHis(SP)_10_L_TEV_-mIFNγ, and Tag-free mIFNγ) produced from infiltrated *N. benthamiana* for 12 h at 37°C with 5% CO_2_ (Busnadiego et al., 2020). Subsequently, the cell monolayer was infected with SINV-eGFP at a multiplicity of infection (MOI) of 1 (Tseng et al., 2015; Jiang et al., 2020). At 24 h post-infection (hpi), the levels of eGFP signals and viral proteins were detected by fluorescence microscopy and IB analysis with specific rabbit primary antibodies. The inhibition ratio of SINV was further determined by ELISA with eGFP-specific antibodies.

### 2.11 Statistical analysis

Statistical analysis was performed using the standard Student’s t-test (SPSS version 20, IBM Corp, Armonk, NY, United States). Mean expression ratio (%) and standard deviation (SD) from three independent experiments with technical triplicates are presented. *p* values < 0.001 were considered significant.

## 3 Results

### 3.1 Design of a cleavable-tag protein expression system based on the BaMV vector

Previous studies have developed several effective fusion tags to increase the productivity and ease of downstream processing (Xu et al., 2007; Xu et al., 2010; Zhang et al., 2016; Ramos-Martinez et al., 2017; Zhang et al., 2019). However, the presence of fusion tags may negatively influence the biological functions of the target proteins, and an effective approach for the removal of such tags is highly desirable. In this study, we designed a series of BaMV-based viral vectors in which cleavable linkers, including L_SrtA_, L_TEV,_ and L_SNAC,_ were inserted between the N-terminal SS^Ext^His(SP)_10_ tag and mIFNγ, as shown in Figure 1A. By using such a BaMV-based secretory expression system, we pursue the possibility of efficient production of near-native recombinant glycoproteins without any foreign fusion tags. To compare the processing efficiency of various cleavable linkers in our BaMV-based vectors, these constructions were infiltrated into *N. benthamiana* leaves through *Agrobacterium*-mediated inoculation, and the expression profiles were further examined. The previously reported constructs of pKB19mIFNγ (Jiang et al., 2019) and pKB19SS^Ext^mIFNγ(SP)_10_ (Jiang et al., 2020) were also included in the experiment, serving as the bases for comparison.

To investigate whether inserted cleavable linkers affect the expression of mIFNγ fusion glycoproteins in the BaMV-based expression system, total proteins extracted from the infiltrated *N. benthamiana* leaf samples were examined at 5 dpi by IB analysis with specific antibodies against mIFNγ or His-tag (Figure 1B). The result of IB analysis using anti-His-tag antibodies revealed the presence of unprocessed SS^Ext^His(SP)_10_L-mIFNγ protein (FP, Figure 1B) and SS^Ext^His(SP)_10_L-mIFNγ glycoprotein (FG, Figure 1B) with relative molecular masses (*M*_*r*_) of 25 kDa and 32–40 kDa, respectively, which are similar to those observed in the control sample SS^Ext^mIFNγ(SP)_10_. The product of FGs (32–40 kDa) was possibly produced by the partial Hyp-O-glycosylation of FPs (25 kDa) via the *N. benthamiana* secretory pathway, as observed previously (Dolan et al., 2014; Jiang et al., 2020). By IB analysis with mIFNγ-specific antibodies, three extra protein bands were detected in leaf extracts of SS^Ext^His(SP)_10_L_TEV_-mIFNγ, with apparent *M*_*r*_ of 16 kDa (Mγ), 18 kDa (1N-MG), and 20 kDa (2N-MG) (indicated at the right of the panel, Figure 1B), as compared to those expressing SS^Ext^His(SP)_10_L_SrtA_-mIFNγ or SS^Ext^His(SP)_10_L_SNAC_-mIFNγ. These extra protein banding patterns were also observed in the control sample SS^Ext^mIFNγ(SP)_10_ (Jiang et al., 2020), which also exhibit the same molecular weight, indicating SS^Ext^His(SP)_10_L_TEV_-mIFNγ glycoproteins might be post-translationally modified by the proteolytic cleavage of the combinational secretory-affinity tag, SS^Ext^His(SP)_10_, with varying degrees of N-glycosylation (including 0N, 1N, and 2N glycosylation, corresponding to the molecular weights of 16 kDa, 18 kDa, and 20 kDa, respectively). It also implied that plant endogenous protease activity could partially digest the FGs to form the various cleaved mIFNγ glycoproteins during the secretory process.

The overall accumulation of various mIFNγ forms was further quantified by ELISA. The result revealed the average levels of TPs produced in leaves infiltrated with *A. tumefaciens* harboring constructs for the expression of SS^Ext^His(SP)_10_L_srtA_-mIFNγ and SS^Ext^His(SP)_10_L_TEV_-mIFNγ accumulated up to 595 ± 60.1 and 688 ± 5.9 μg/g fresh weight, accounting for 9.8% and 10.8% of the TSP, respectively (Table 1), which are significantly higher than those observed in the control groups. The result indicated that the fusion of combinational secretory-affinity tag (SS^Ext^His(SP)_10_L_srtA_ or SS^Ext^His(SP)_10_L_TEV_) could further improve the yield of TPs. However, the expression of SS^Ext^His(SP)_10_L_SNAC_-mIFNγ glycoprotein in infiltrated *N. benthamiana* leaves was lower than that of other constructs (Figure 1B; Table 1), and thus it was excluded in the following experiments.

**TABLE 1:** Expression levels of TPs as quantified by ELISA.

	Yield (mg/kg FW)	TSP (mg/kg)	%TSP
mIFNγ	80^a^ ± 10.6	5449 ± 397	1.5
SS^Ext^mIFNγ(SP)_10_	485^b^ ± 65.5	5637 ± 157	8.6
SS^Ext^His(SP)_10_L_SrtA_-mIFNγ	595^c^ ± 60.1	6047 ± 241	9.8
SS^Ext^His(SP)_10_L_TEV_-mIFNγ	688^c^ ± 5.9	6380 ± 214	10.8
SS^Ext^His(SP)_10_L_SNAC_-mIFNγ	462^b^ ± 44.1	6001 ± 76	7.7

Statistical analyses were performed using ANOVA. Mean values with dissimilar superscripts (a, b, and c) are significantly different at the level of *p*-value <0.001. Abbreviations: FW, fresh weight; TSP, total soluble protein.

### 3.2 Proteolytic cleavage of SS^Ext^His(SP)_10_L_SrtA_-mIFNγ using sortase A

**FIGURE 2:**
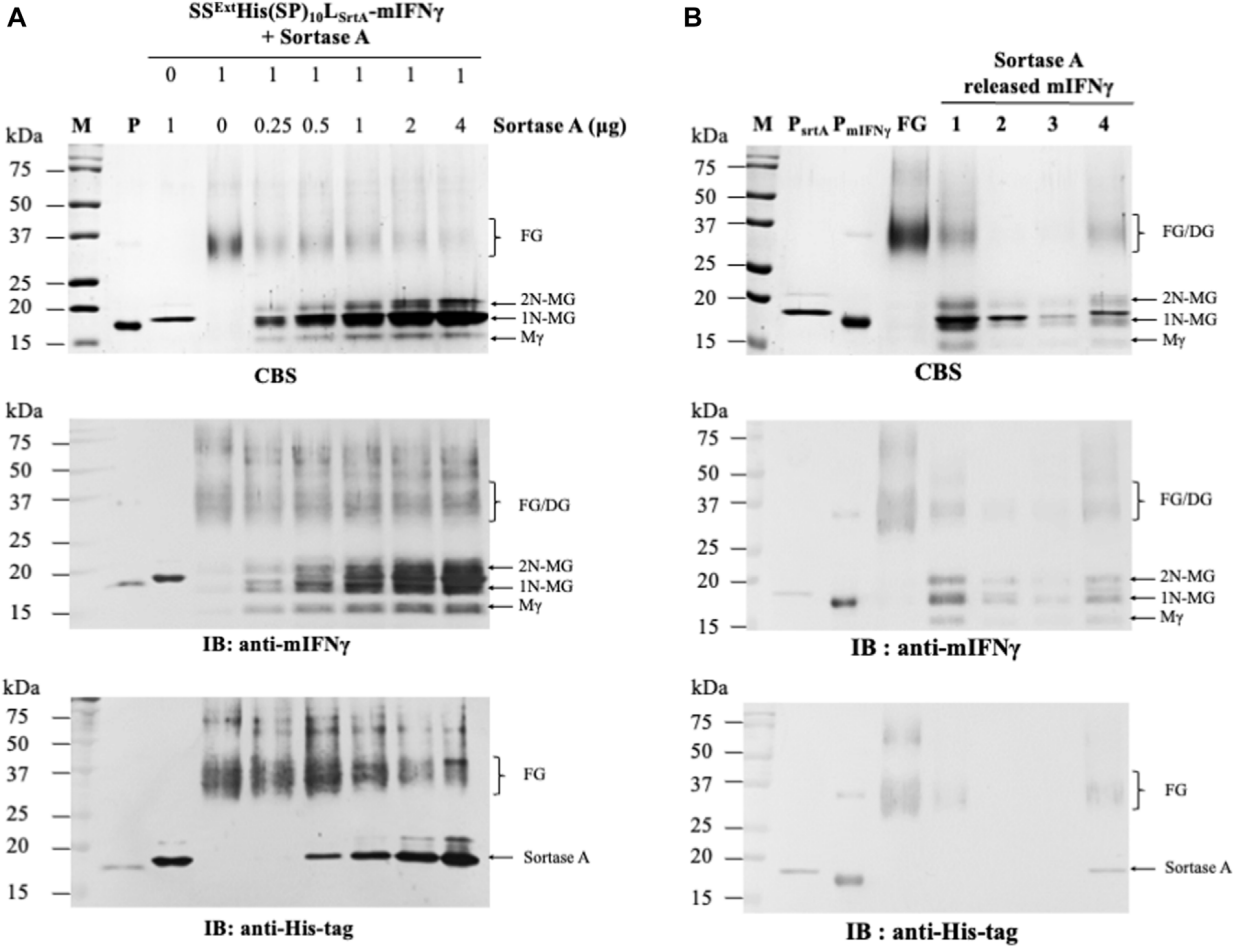
Processing of SS^Ext^His(SP)_10_L_SrtA_-mIFNγ glycoproteins by SrtA protease. **(A)** Optimization of the reaction condition. To determine the optimal protein-to-enzyme ratio, the purified FGs (1 μg, SS^Ext^His(SP)_10_L_SrtA_-mIFNγ) were incubated with different concentrations of SrtA, as indicated in the panel, at 28°C overnight. Subsequently, each reaction was analyzed by SDS-PAGE and visualized with CBS and IB analysis with antibodies specific to mIFNγ or His-tag. **(B)** Purification of SrtA-released mIFNγ from SS^Ext^His(SP)_10_L_SrtA_-mIFNγ glycoproteins by the Ni^2+^-NTA column. Each fraction was collected and assayed by SDS-PAGE, followed by CBS and IB analysis with specific antibodies, as indicated at the bottom of the panel. M, marker; P, positive control, purified mIFNγ protein derived from *E. coli*; FG, SS^Ext^His(SP)_10_L_SrtA_mIFNγ glycoprotein; lane 1, total reaction product containing released mIFNγ glycoproteins from FGs (1 μg, SS^Ext^His(SP)_10_L_SrtA_-mIFNγ); lane 2, flow-through fraction; lane 3, eluted fraction using 25 mM imidazole; lane 4, eluted fraction using 300 mM imidazole. The positions of different forms of mIFNγ or sortase A are indicated on the right of each panel. Mγ, M mIFNγ; 1N-MG, M monoglycosylated mIFNγ; 2N-MG, M diglycosylated mIFNγ; DG, dimeric mIFNγ glycoprotein.

To determine the feasibility of SrtA in removing the combinational secretory-affinity tag from the SS^Ext^His(SP)_10_L_srtA_-mIFNγ fusion glycoproteins (FGs), the following experiment was performed. Ni^2+^-NTA column-purified FGs (32–40 kDa) and SrtA (17.4 kDa) were collected, examined by SDS-PAGE (Supplementary Figure S1), and quantified for their total soluble protein (TSP) levels by using the Bradford colorimetric assay (Sigma-Aldrich, St. Louis, MO, United States). To determine the optimal protein-to-enzyme ratio, the purified FGs (1 μg, SS^Ext^His(SP)_10_L_SrtA_-mIFNγ) were incubated with various concentrations of SrtA (two-fold serial dilutions from 4 to 0.25 μg/μL). Subsequently, each reaction was analyzed by SDS-PAGE, and the FGs or various cleaved mIFNγ forms were visualized by CBS and IB analysis with specific antibodies against mIFNγ or His-tag. As shown in the CBS-stained gel, the cleaved mIFNγ forms with Mγ (16 kDa), 1N-MG (18 kDa), and 2N-MG (20 kDa) were partially released from FGs (SS^Ext^His(SP)_10_L_SrtA_-mIFNγ) following SrtA digestion (Figure 2A). Additionally, the dimeric and heterogeneous mIFNγ glycoproteins (32–40 kDa, DG) were also observed, which might be generated from the subsequent dimerization of two cleaved mIFNγ monomers with different degrees of N-glycosylation (Sareneva et al., 1994; Sareneva et al., 1995). The result of IB analysis with mIFNγ-specific antibodies showed that the maximal efficiency for the removal of combinational secretory-affinity tag was approximately 50%, with high doses (2 and 4 μg/μL) of SrtA digestion. Analysis with His-tag-specific antibodies further verified that the cleavage of FGs was not complete (Figure 2). Based on the above observations, a 1:2 ratio of FG to enzyme was chosen for SrtA-mediated cleavage. The cleaved combinational secretory-affinity tag was subsequently separated from the target proteins by passing through an Ni^2+^-NTA column. However, analysis of the eluted fraction from 300 mM imidazole treatment revealed that the majority of the processed mIFNγ glycoproteins (32–40 kDa), including Mγ (16 kDa), 1N-MG (18 kDa), and 2N-MG (20 kDa), were still accompanied with SrtA (Figures 2B, IB panel, lane 4). This observation indicated that SrtA-mediated digestion could not efficiently process the FGs (SS^Ext^His(SP)_10_L_SrtA_-mIFNγ) and that the contamination of the combinational secretory-affinity tag and SrtA may pose a threat to the downstream process of target proteins.

### 3.3 Proteolytic cleavage of SS^Ext^His(SP)_10_L_TEV_-mIFNγ using TEV protease

To test the applicability of L_TEV_ linker and TEV protease in the BaMV-based system, experiments similar to those described above were performed. Both SS^Ext^His(SP)_10_L_TEV_-mIFNγ and TEV proteins were also initially purified by the Ni^2+^-NTA column. Analysis of the eluates revealed that the FGs (32–40 kDa) could be efficiently obtained, accompanied with a few processed mIFNγ forms, including Mγ (16 kDa), 1N-MG (18 kDa), and 2N-MG (20 kDa) (Supplementary Figure S2).

**FIGURE 3:**
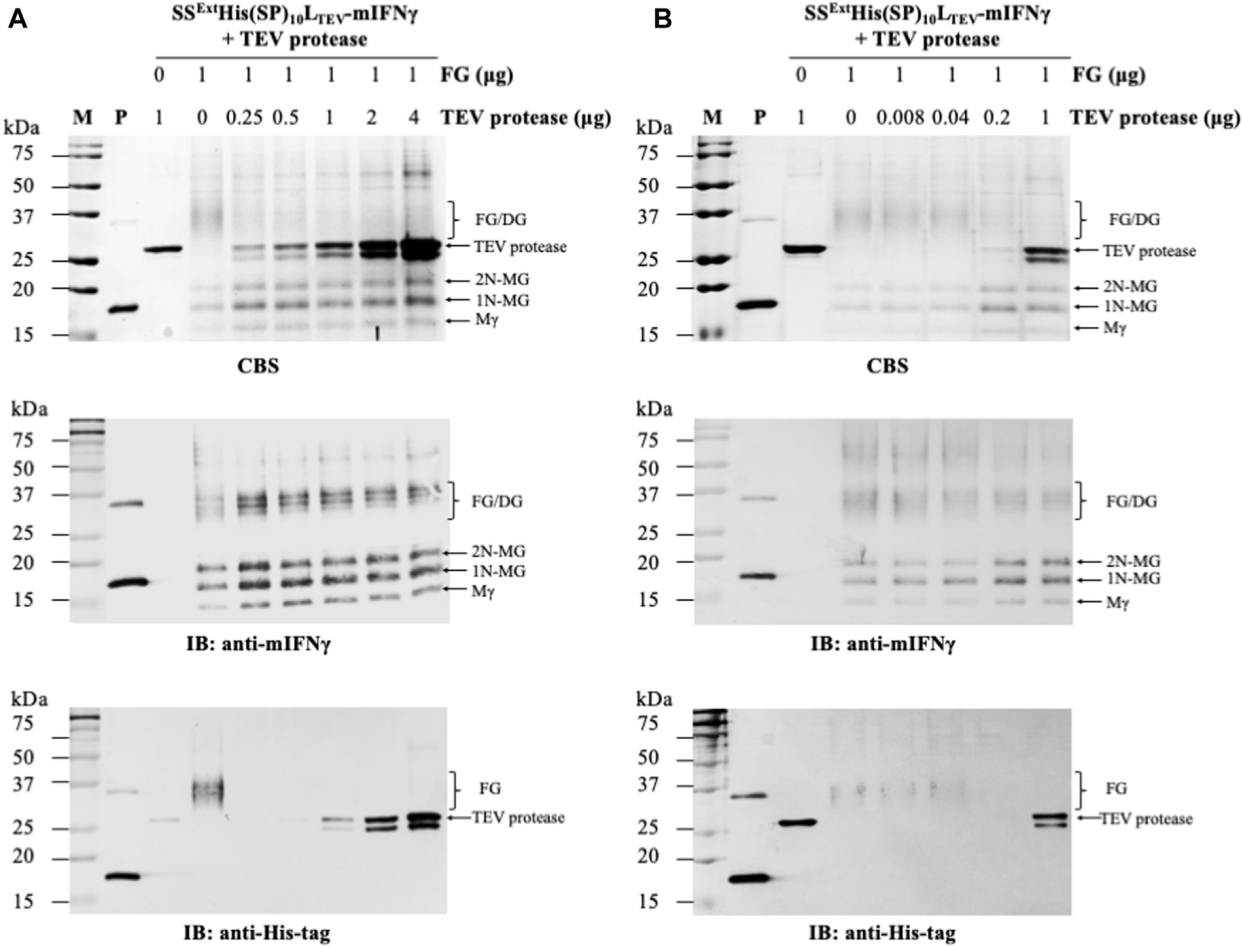
Processing of SS^Ext^His(SP)_10_L_TEV_-mIFNγ glycoproteins by TEV protease. To determine the optimal protein-to-enzyme ratio, the purified FGs (1 μg, SS^Ext^His(SP)_10_L_SrtA_-mIFNγ) were initially incubated with 2-fold serial dilutions of TEV proteinase (starting from 4 μg/μL) **(A)** and further fine-tuned with 5-fold serial dilutions of TEV proteinase (starting from 1 μg/μL) **(B)**, as indicated in the panel, at 4°C overnight. Subsequently, each reaction was assayed by SDS-PAGE, followed by CBS and IB analysis, as described above. M, marker; P, positive control, purified mIFNγ protein derived from *E. coli*; FG, SS^Ext^His(SP)_10_L_TEV_-mIFNγ glycoprotein. The positions of different forms of mIFNγ are indicated on the right of each panel. Mγ, M mIFNγ; 1N-MG, M monoglycosylated mIFNγ; 2N-MG, M diglycosylated mIFNγ; DG, dimeric mIFNγ glycoprotein.

To determine the optimal reaction condition, the purified SS^Ext^His(SP)_10_L_TEV_-mIFNγ glycoproteins (1 μg) were treated with different concentrations of TEV protease (two-fold serial dilutions ranging from 4 to 0.25 μg/μL) at 4°C overnight. Subsequently, each reaction was analyzed by SDS-PAGE, and the FGs or the cleaved mIFNγ forms were visualized by CBS and IB. The result of IB analysis with His-tag-specific antibodies (Figure 3A, the bottom panel) showed that the SS^Ext^His(SP)_10_ tag was completely removed by TEV protease from FGs (SS^Ext^His(SP)_10_L_TEV_-mIFNγ). To further fine-tune the reaction condition for the removal of the SS^Ext^His(SP)_10_ tag from FGs, the purified SS^Ext^His(SP)_10_L_TEV_-mIFNγ glycoproteins (1 μg) were further incubated with five-fold serial dilutions (ranging from 1 μg/μL to 0.008 μg/μL) of TEV protease. Analysis of the digestion result by IB with His-tag-specific antibodies revealed that FGs could be efficiently digested by using 0.2 μg/μL of TEV protease (Figure 3B, the bottom panel). Consequently, a 1:0.2 ratio of FG to enzyme was chosen for TEV protease-mediated cleavage. As expected, following the processing of the fusion tag, the monomeric polypeptide subunit of mIFNγ glycoproteins would assemble into dimeric and heterogeneous mIFNγ glycoproteins (32–40 kDa, DG), as shown by IB analysis with anti-mIFNγ-specific antibodies (Figures 3A, B, the middle panel). These findings indicated that TEV protease may serve as an efficient process in the production of target proteins containing L_TEV_-linked fusion tags in the BaMV-based expression vector.

**FIGURE 4:**
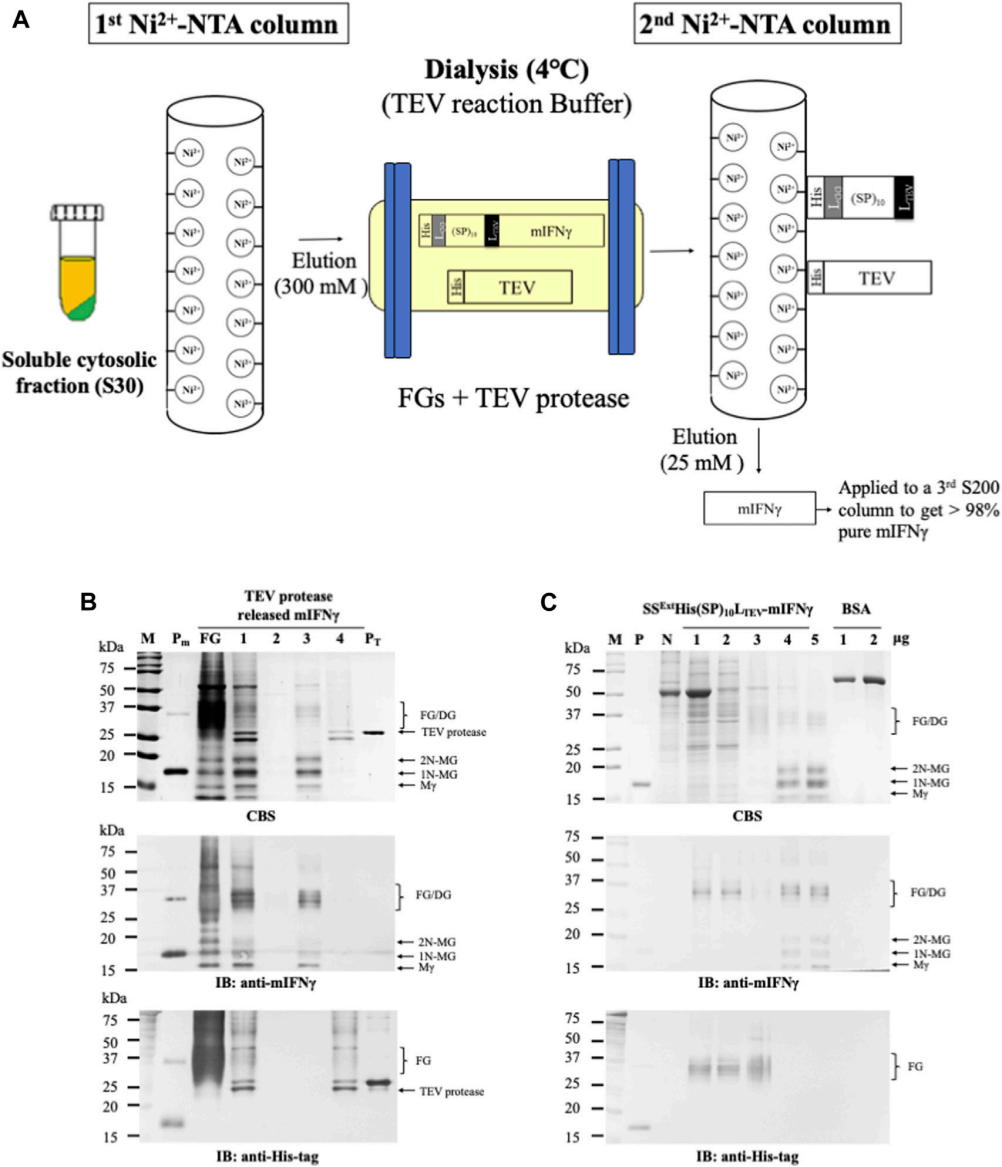
Purification of TEV protease-released mIFNγ from SS^Ext^His(SP)_10_L_TEV_-mIFNγ glycoproteins by Ni^2+^-NTA and gel filtration chromatography. **(A)** Schematic representation of the process for purification of tag-free mIFNγ glycoproteins. Briefly, the target fusion proteins containing His-tags were initially purified through an immobilized metal affinity chromatography (IMAC) column. Following incubation with TEV protease in a dialysis tubing overnight, the released tag-free mIFNγ glycoproteins were further purified through a second IMAC column. **(B)** Analysis of the released mIFNγ glycoproteins. The FGs (10 mg, SS^Ext^His(SP)_10_L_TEV_-mIFNγ) were processed using TEV protease (2 mg)-mediated cleavage, followed by purification through a Ni^2+^-NTA column. Each fraction was collected and assayed by SDS-PAGE, followed by CBS and IB analysis, as described above. Lane 1, total reaction content containing FGs; lane 2, flow-through; lane 3, eluted fraction of tag-free mIFNγ glycoproteins using 25 mM imidazole; lane 4, eluted fraction of TEV protease using 300 mM imidazole (lane 4). **(C)** SDS-PAGE analysis of each fraction from the three-step chromatography purification process. Each fraction was collected and assayed by SDS-PAGE, followed by CBS and IB analysis, as described above. Lane 1, soluble fraction following centrifugation (S30); lane 2, S30 treated with acetic acid (S30t); lane 3, eluted fraction from first Ni^2+^-NTA purification; lane 4, eluted fraction from the second Ni^2+^-NTA purification; lane 5, products of the third gel filtration purification. M, marker; P, positive control, purified mIFNγ protein derived from *E. coli*; FG, SS^Ext^His(SP)_10_L_TEV_-mIFNγ glycoproteins; Mγ, M mIFNγ; 1N-MG, M monoglycosylated mIFNγ; 2N-MG, M diglycosylated mIFNγ; DG, dimeric mIFNγ glycoprotein; BSA, bovine serum albumin as a protein loading standard.

Subsequently, we developed a protocol for obtaining tag-free recombinant glycoproteins from plant-made SS^Ext^His(SP)_10_L_TEV_-mIFNγ, as illustrated in Figure 4A. To examine whether this protocol could be efficiently scaled up for processing the FGs into tag-free mIFNγ, 10 mg of SS^Ext^His(SP)_10_L_TEV_-mIFNγ was incubated with TEV protease (2 mg/mL) in a dialysis tube (MWCO 12–14 kDa) overnight at 4°C. After incubation, the released mIFNγ glycoproteins and SS^Ext^His(SP)_10_ tag were separated by passing through a second Ni^2+^-NTA column. Analysis of each fraction by SDS-PAGE and IB with mIFNγ-specific antibodies revealed that the released mIFNγ glycoproteins, including Mγ (16 kDa), 1N-MG (18 kDa), 2N-MG (20 kDa), and DG (32–40 kDa), were efficiently recovered by a low concentration of imidazole (25 mM) (Figure 4B, the middle panel, lane 3). The result of IB analysis with His-tag-specific antibodies confirmed that the combinational secretory-affinity tag was efficiently removed from FGs (Figure 4B, the bottom panel, lane 3). Additionally, TEV protease (*M*_*r*_ of 27 kDa) was found in the fractions eluted with 300 mM imidazole (Figure 4B, lane 4), indicating that TEV protease could be separated from the products and recovered for recycling through the Ni^2+^-NTA column. These results showed that the use of TEV protease-mediated processing could be scaled up to efficiently remove the SS^Ext^His(SP)_10_ tag from SS^Ext^His(SP)_10_L_TEV_-mIFNγ. The observation also suggested that this system could be used as a means to mass-produce near-native mIFNγ glycoproteins and other recombinant therapeutic proteins. To further improve the purity of tag-free mIFNγ, the collected fractions were pooled and applied to gel filtration chromatography (Superdex™ 200 pg, S200). Each step-fraction was collected, and the protein contents were confirmed again by SDS-PAGE stained with CBS and IB with mIFNγ-specific antibodies (Figure 4C). The results indicated that tag-free mIFNγ could be efficiently obtained. The final yield was estimated to be approximately 102 ± 3 mg/kg fresh tissue weight (FW) with a purity higher than 98%, which is comparatively higher than that for SS^Ext^mIFNγ(SP)_10_ (94 ± 7 mg/kg FW), as reported previously (Jiang et al., 2020).

### 3.4 Efficacy of IFN-γ variants against chimeric *Sindbis virus*

**FIGURE 5:**
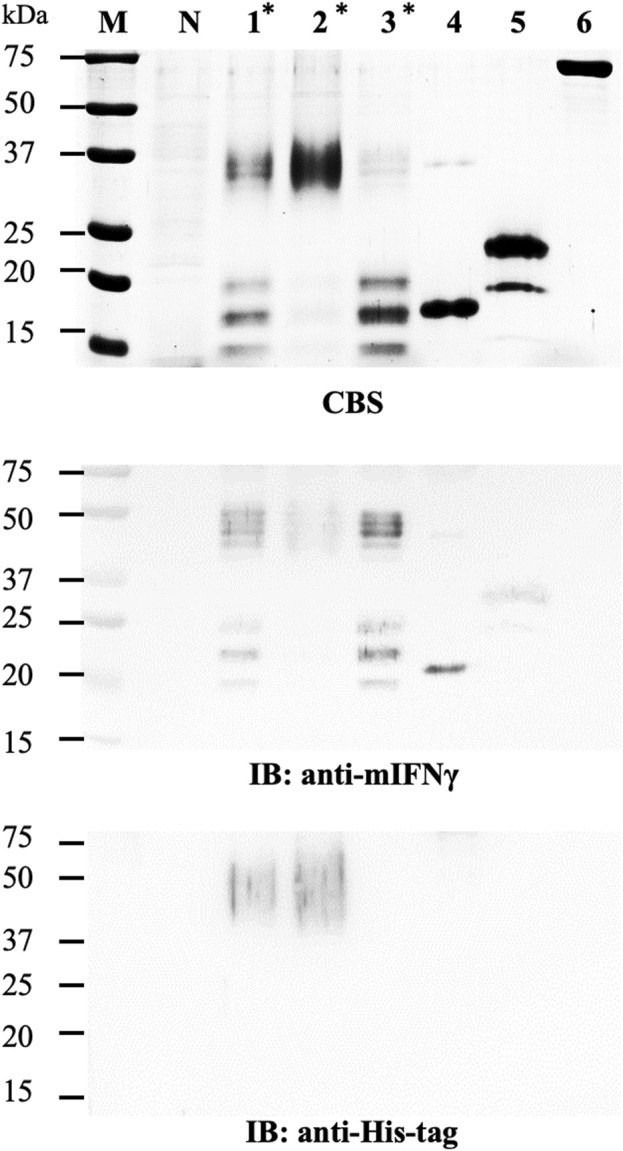
Comparison of various hIFNγ variants. Each IFNγ variant (1 μg) was analyzed by SDS-PAGE, followed by CBS and IB analysis, as described above. Lane 1, fusion protein SS^Ext^mIFNγ(SP)_10_ from *N. benthamiana* leaf homogenates; lane 2, fusion SS^Ext^His(SP)_10_L_TEV_-mIFNγ glycoproteins; lane 3, tag-free mIFNγ glycoprotein; lane 4, commercial *E. coli* expressed IFNγ; lane 5, HEK293-expressed IFNγ; lane 6, BSA as the protein loading standard. M, marker; *, mIFNγ variants collected following the three-step purification protocol described above.

**FIGURE 6:**
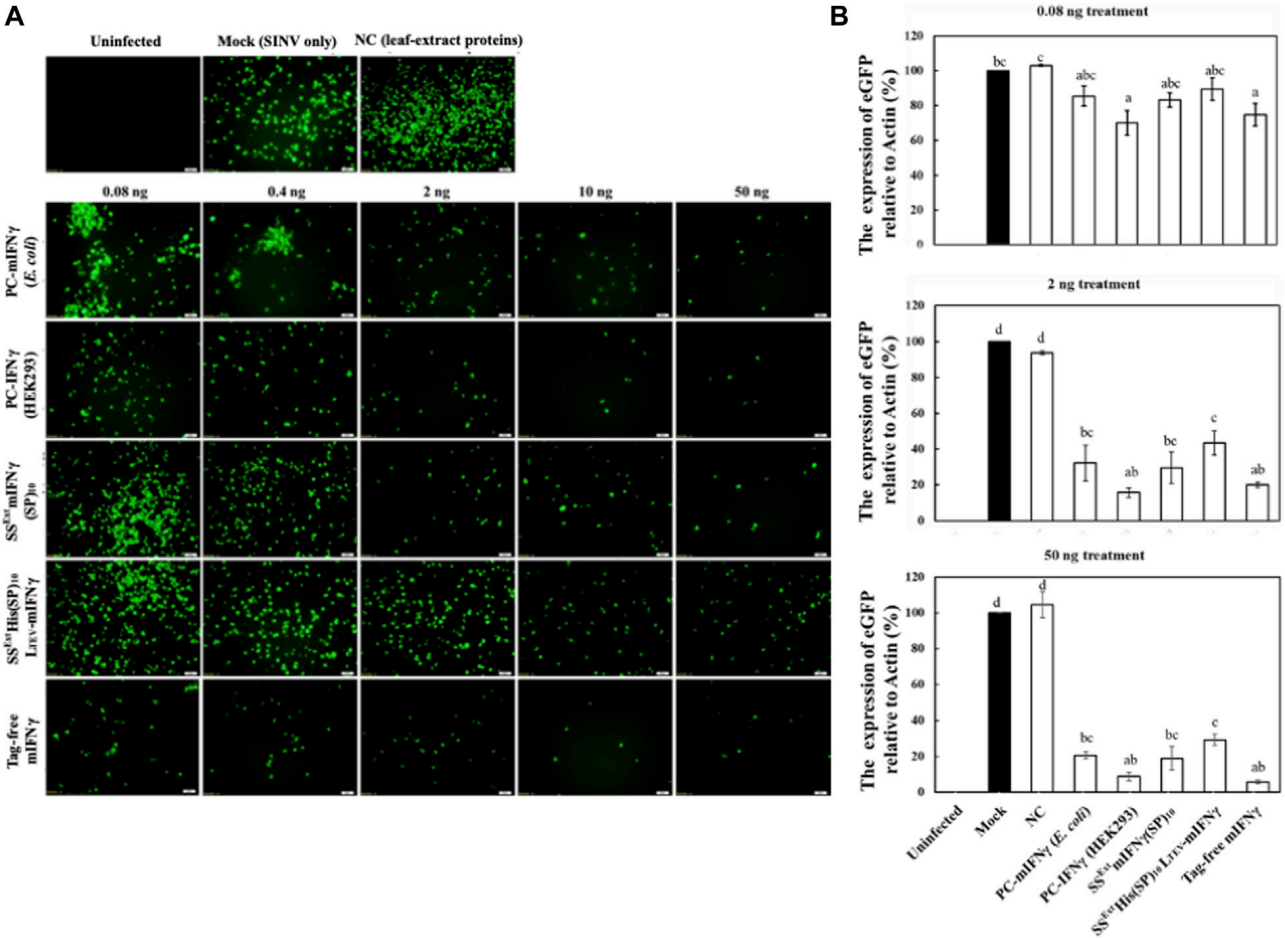
Effect of fusion tags on biological activity of mIFNγ against SINV. HEK293-T cells were left uninfected or pre-treated with DMEM (Mock), 50 ng of leaf-extract proteins as the negative control (NC), different concentrations of commercially available IFNγ proteins as positive controls (PCs), designated as PC-mIFNγ (produced from *E. coli*), PC-IFNγ (produced from HEK293), or various recombinant proteins produced from plants, including SS^Ext^mIFNγ(SP)_10_, SS^Ext^His(SP)_10_L_TEV_-mIFNγ, or tag-free mIFNγ for 12 h. Subsequently, cells were infected with the reporter virus, SINV-eGFP, at an MOI of 1 for 24 h. The accumulated eGFP signals were directly observed by fluorescence microscopy **(A)** (scale bar = 50 μm) and quantified by densitometry **(B)**. The eGFP level in DMEM (Mock)-treated cells was arbitrarily set as 100%, and relative protein accumulation levels in other samples were estimated. Statistical analysis was performed using one-way ANOVA with Tukey’s *post hoc* multiple comparison analysis. The *p*-value of <0.05 was considered significantly different, as denoted by different letters.

**FIGURE 7:**
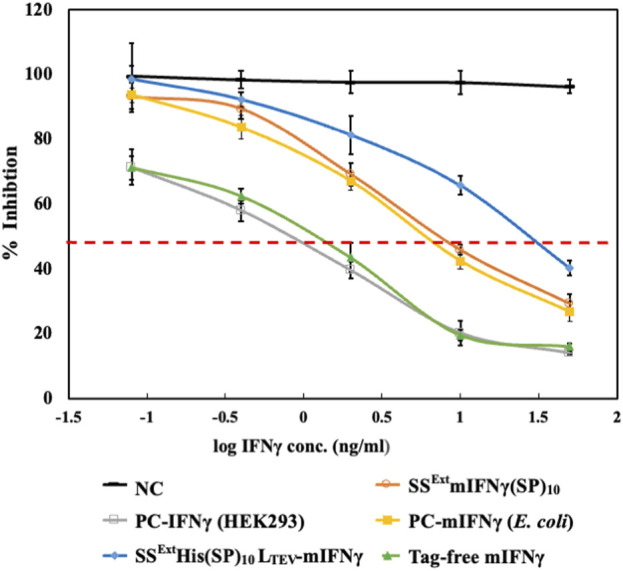
Relative anti-SINV activity of various interferon gamma variants. The relative inhibition ratio (%) of SINV in cells treated with different samples was estimated using the quantification of the eGFP level by ELISA. The dashed line in red indicates 50% of the relative inhibition. The half-maximal inhibitory concentration (IC_50_) of each sample is represented in Table 2.

The inhibitory effect of recombinant IFN-γ has been demonstrated against several viruses (Parvez et al., 2006; Wang et al., 2014; Busnadiego et al., 2020) and cancer cells (Razaghi et al., 2017; Shen et al., 2018). Moreover, as presented in our previous study, plant-made SS^Ext^mIFN(SP)_10_ was shown to induce antiviral activity in a reporter *Sindbis virus* expressing eGFP (SINV-eGFP) and reduce influenza virus (H1N1 strain) replication to a similar extent, as achieved by the commercial mIFNγ produced in *E. coli* (Jiang et al., 2020). To evaluate the efficacy of the IFNγ variants, including SS^Ext^mIFNγ(SP)_10_, SS^Ext^His(SP)_10_L_TEV_-mIFNγ, and tag-free mIFNγ purified from *N. benthamiana,* the recombinant proteins were prepared in PBS buffer and the concentrations of each sample were further confirmed by SDS-PAGE stained with CBS and IB with mIFNγ-specific or His-tag-specific antibodies (Figure 5). The commercial mIFNγ produced in *E. coli* or mammalian cells, designated PC-mIFNγ (*E. coli*) and PC-IFNγ (HEK-293), respectively, were used as the positive controls. The antiviral effect was then evaluated using the SINV-eGFP reporter virus as a target pathogen. HEK293 cells were cultured in DMEM with IFN-γ variants at various concentrations, which were 5-fold serially diluted from 50 to 0.08 ng/mL for 12 h, followed by challenging with the SINV-eGFP. The cells cultured in DMEM treated with PBS buffer were used as the mock treatment group (Mock), whereas those treated with the total protein extract of healthy *Nicotiana benthamiana* served as the negative control (NC). At 24 h post-infection (hpi), the eGFP fluorescence, representing the overall infection status, was examined and recorded by fluorescent microscopy. As shown in Figure 6A, upon treatment with 2–50 ng IFNγ variants, the eGFP fluorescence signals were obviously decreased and mostly confined to a single-cell level, as compared to those in the Mock and NC (50 ng) groups. It is worth noting that cells treated with the tag-free mIFNγ variant at low doses (0.08 ng or 0.4 ng) had lower eGFP signals, demonstrating the antiviral efficacy of tag-free mIFNγ. The results of eGFP fluorescence intensity measurements and IB analysis showed that treatment with the tag-free mIFNγ variant at the lowest dose (0.08 ng) significantly decreased the level of eGFP (*p-*values <0.001), which is comparable to the effect of commercial PC-IFNγ (HEK293). Treatments with three IFNγ variants at medium (2 ng) or high (50 ng) doses reduced the eGFP levels in a dose-dependent manner (Figure 6B; Supplementary Figure S3). Among them, the tag-free mIFNγ was observed to have a higher efficacy in decreasing SINV-eGFP infection as compared to those of variants with fusion tags, including SS^Ext^mIFNγ(SP)_10_ and SS^Ext^His(SP)_10_L_TEV_-mIFNγ (a significant difference with *p-*values <0.001). To corroborate this result, the inhibition ratio of SINV-eGFP infection was determined by ELISA for quantification of eGFP levels, and the half-maximal inhibitory concentration (IC_50_) of each recombinant protein was estimated. As shown in Figure 7; Table 2, tag-free mIFNγ exhibited a higher inhibitory effect on SINV-eGFP infection, which was similar to that of commercial PC-IFNγ (HEK293), with IC_50_ values of 2.5–2.9 ng/mL. In contrast, the IC_50_ values of SS^Ext^mIFNγ(SP)_10_ and SS^Ext^His(SP)_10_L_TEV_-mIFNγ were 7.9 and 37.2 ng/mL, respectively, which were notably higher than that of the tag-free protein. The lower IC_50_ value and similar efficacy comparable with commercial IFNγ (HEK-293) protein indicated that plant-made tag-free mIFNγ exhibits satisfactory antiviral activity, which also suggested that these IFNγ glycoproteins may maintain the native folding suitable for inhibiting viruses.

**TABLE 2:** Half-maximal inhibitory concentration (IC50) of each sample.

Protein sample	IC50 (ng/mL)
NC	-
PC-IFNγ (E.coli)	8.6
PC-IFNγ (HEK293)	2.5
SSExtmIFNγ(SP)10	7.9
SSExtHis(SP)10LTEV-mIFNγ	37.2
Tag-free mIFNγ	2.9

## 4 Discussion

The safety and productivity of therapeutic proteins are among the major considerations for the biomedical application of plant-made pharmaceuticals. In this study, we have developed an improved BaMV-based expression system using the combinational secretory-affinity tag attached to the mIFNγ protein through various cleavable linkers (L_SrtA_, L_TEV_, and L_SNAC_) and tested the efficiency for the removal of fusion tags from FGs. We demonstrated that the TEV protease effectively processed L_TEV_-linked FGs to release tag-free mIFNγ glycoproteins, containing Mγ (16 kDa), 1N-MG (18 kDa), and 2N-MG (20 kDa), which would undergo self-assembly to form the biologically active DG form (32–40 kDa). Both the cleaved combinational secretory-affinity tag and TEV protease were efficiently removed through a second immobilized metal affinity and gel filtration chromatography (IMAC) for obtaining the tag-free forms of mIFNγ glycoproteins. In addition, the antiviral activity of mIFNγ glycoproteins was verified and shown to exhibit similar or higher biological activities against the *Sindbis viru*s as compared to those of the commercial HEK293-expressed hIFNγ or tagged versions of mIFNγ. These observations highlight the potential of this refined BaMV-based expression vector as an efficient system for the production of plant-made biopharmaceutical candidates with antiviral therapeutic efficacy.

Recently, several groups have successfully established plant-based protein secretion systems based on HypGP tag techniques (Xu et al., 2007; Xu et al., 2010; Zhang et al., 2016; Ramos-Martinez et al., 2017; Zhang et al., 2019). The expression of secreted fusion proteins either in inoculated plant leaves or suspension cell culture by fusion of the combinational secretory peptides, including plant-derived SS and “Ser-Pro” motif repeats to target proteins, provides feasible production for large-scale downstream processing. Additional fusion of affinity His-tag to the target protein is a useful technique for the efficient purification of target proteins (Wilken and Nikolov, 2012). However, there has been no attempt in these previous studies to remove the combinational secretory-affinity tag from FGs through enzymatic or chemical cleavage.

Our previous study showed that the proteolytic cleavage of N- and C-terminal fusions of SS^Ext^mIFNγ(SP)_10_ through plant secretory processes may convert the FGs into the maturation forms of mIFNγ glycoproteins, including Mγ (16 kDa), 1N-MG (18 kDa), 2N-MG (20 kDa), and DG (32–40 kDa) (Jiang et al., 2020). However, the native processing activity of the plant secretory system was not sufficient to produce tag-free mIFNγ efficiently. In this study, it was noted that the SS^Ext^mIFNγ(SP)_10_ containing 6X His-tag at the C-terminus was still detected by His-tag-specific antibodies (Figure 1B, the bottom panel), indicating that FGs were only partially processed. Therefore, various cleavable linkers, including L_SrtA_, L_TEV_, and L_SNAC_, were taken into consideration for the efficient production of tag-free mIFNγ in *N. benthamiana* through the BaMV-based vector in this study. We showed that all three cleavable linkers allowed the successful expression of FGs by the renovated BaMV-based vector, with partial O-glycosylated proteins of estimated molecular masses ranging from 32 to 40 kDa (Figure 1B, the bottom panel). The yield is similar to those observed in *N. benthamiana* expressing SS^tab^ (SP)_32_-EGFP (Dolan et al., 2014) and in tobacco hairy root expressing SS^tab^ (SP)_32_-EGFP (Zhang et al., 2019). Among these constructs with different inserted linkers, the expression level of SS^Ext^His(SP)_10_L_SNAC_-mIFNγ was lower than those of SS^Ext^His(SP)_10_L_SrtA_-mIFNγ and SS^Ext^His(SP)_10_L_TEV_-mIFNγ (Figure 1B, the bottom panel), suggesting that different cleavable linkers may affect the expression, conformational flexibility, and/or stability of FGs. It is worth noting that SS^Ext^His(SP)_10_L_TEV_-mIFNγ glycoproteins were partially cleaved to Mγ (16 kDa), 1N-MG (18 kDa), and 2N-MG (20 kDa) by endogenous proteases in plants, possibly through the secretory pathway, which was in agreement with our previous result for SS^Ext^mIFNγ(SP)_10_ expressed from the secretory BaMV-based vector (Jiang et al., 2020). Similar observations of this proteolytic process have also been reported (Zhang et al., 2011; Puchol Tarazona et al., 2021). It has been shown that plant endogenous Kex2p-like serine protease or additional unknown protease may cleave the GMCSF-L-GFP protein (FP) *in vivo* to release the individual protein through the plant secretory pathway (Zhang et al., 2011). Additionally, two apoplastic-abundant subtilisin-like serine proteases, NbSBT1 and NbSBT2, were recently identified to be responsible for the major proteolytic processes on recombinant IgG1 glycoproteins during protein secretion in *N. benthamiana* (Puchol Tarazona et al., 2021). Although these reports demonstrated that the fusion tags on target proteins may be automatically removed by endogenous proteolytic processing, especially through the secretory pathways, the efficiencies seem to require further improvement for industrial-scale production. Furthermore, the yield of the target proteins may be affected by the addition of different fusion tags. To ensure the efficient production of tag-free target proteins, various chemically or enzymatically cleavable linkers are used in the expression constructs. The above observations indicated that it is necessary to test the effects on yield and cleavage efficiency of such linkers for optimization of protein expression systems. For this purpose, the BaMV-based tag-free expression vector system developed in this study provides three cleavable linkers in the construct that may exert different effects on different target proteins and thus could be conveniently tested and optimized for different needs.

Fusion tags are often used to increase the efficiency of downstream production since they can be helpful for enhancing the stability and solubility of target proteins as well as their purification processes (Arnau et al., 2006; Yadav et al., 2016; Liu and Timko, 2022). The use of a combinational secretory-affinity tag in this study was not only for the isolation of soluble FGs in the S30t fraction but also the successful recovery of pure SS^Ext^His(SP)_10_L_SrtA_-mIFNγ and SS^Ext^His(SP)_10_L_TEV_-mIFNγ glycoproteins after the primary Ni^2+^-NTA purification (Supplementary Figure S1, 2). Although the fusion technology is a powerful tool for the efficient production of many recombinant proteins, previous studies have shown that fusion tags would affect the biological function and immunogenicity of recombinant proteins through interference with the formation of native structures, especially the dimer/oligomer conformation (Gaberc-Porekar et al., 1999; Wu and Filutowicz, 1999; Yadav et al., 2016). The removal of fusion tags from FGs using enzymatic or chemical cleavage is thus a critical procedure for producing therapeutic proteins for biomedical use (Yadav et al., 2016; Dang et al., 2019; Islam et al., 2019). The enzyme-mediated proteolytic cleavage assays in this study showed that the TEV protease could near-completely remove the combinational affinity tag from FGs (Figure 3) as compared to those of SrtA-mediated cleavage (Figure 2). Additionally, under biocompatible conditions at 4°C overnight, TEV protease was active to cleave the FGs to release C-terminal mIFNγ glycoproteins, including Mγ (16 kDa), 1N-MG (18 kDa) and 2N-MG (20 kDa), resulting in a biologically active and heterogeneous DG form (32–40 kDa) with multiple bands by self-assembly. Subsequently, the combinational secretory-affinity tag and TEV protease could be efficiently removed through a second Ni^2+^-NTA column to obtain tag-free mIFNγ glycoproteins (Figure 4B, lane 3). After further gel filtration purification, tag-free mIFNγ glycoproteins could be obtained successfully, with a higher yield as compared to that of SS^Ext^mIFNγ(SP)_10_ glycoproteins reported previously (Jiang et al., 2020). The result demonstrated that the combinational fusion tags, either SS^Ext^His(SP)_10_L_SrtA_ or SS^Ext^His(SP)_10_L_TEV_, N-terminally fused to mIFNγ was advantageous to the increase of productivity for downstream purification processes.

The current procedure for the production of tag-free mIFNγ might be too tedious for further industrial applications. A streamlined process might be developed as a one-column-for-all format as shown in Supplementary Figure S4: the plant-made fusion proteins are initially bound on the Ni^2+^-NTA column and separated from the non-target proteins. Following in-column cleavage of the fusion protein with TEV protease, which also contains the 6X His-tag, the tag-free mIFNγ target protein is then eluted and collected in pure form. In the final elution step, the TEV containing 6X-His-tag may be collected and reused in the subsequent processes. In this streamlined procedure, only one Ni^2+^-NTA column is used, and the TEV cleavage step is performed in the column, which may significantly reduce the required resources and processes as compared to the procedure shown in Figure 4A.

Glycosylation is one of the major post-translational modifications of proteins that occurs during ER and Golgi processing within eukaryotic cells, resulting in the addition of different glycans on transported proteins (Faye et al., 2005; Gomord et al., 2010; Liu and Timko, 2022). The native mIFNγ has two glycosylation sites which are known to be required for resistance to granulocyte proteases, cathepsin G, and elastase and play an important role in increasing hIFNγ half-life in human blood (Abiri et al., 2016; Cao et al., 2022). In our previous study, glycosylation of target protein in plant cells has been achieved by fusing plant-specific SS peptide to a recombinant hIFNγ, leading to successful production of glycoprotein with diverse complex-type N-glycans (Jiang et al., 2020). It has been reported that the degrees of similarity in glycosylation patterns between native hIFNγ and those produced in different expression systems are in the order of native hIFNγ (HEK293 cell) > mammalian cells (CHO) > plant cells (*N. benthamiana*) > insect cells (Sf9) > yeast (*Pichia pastoris*) > prokaryotic cells (*E. coli*) (Abiri et al., 2016). In this study, plant-made mIFNγ glycoproteins without undesired tags were shown to induce a higher antivirus activity against SINV-eGFP, as compared to those of the other fusion variants, including SS^Ext^mIFNγ(SP)_10_, SS^Ext^His(SP)_10_L_TEV_-mIFNγ, and PC-mIFNγ (*E. coli*) (Figure 6; Figure 7; Table 2). This observation indicated that glycosylated, tag-free mIFNγ could enhance therapeutic efficacy similar to those induced by HEK293- and CHO-expressed hIFNγ with a complex-type glycan that exhibits higher levels of therapeutic efficacy against ovarian cancer cells as compared to its non-glycosylated form (Razaghi et al., 2017).

## 5 Conclusion

In this study, we have renovated the BaMV-based vector system for successful removal of the combinational secretory-affinity tag from FGs for the production of tag-free glycoproteins in *N. benthamiana*. We demonstrated that, through fusing the combinational secretory-affinity (SS^Ext^His(SP)_10_) tag and TEV-cleavable peptide (L_TEV_) to the N-terminal of the mIFNγ protein, rapid accumulation of soluble SS^Ext^His(SP)_10_L_TEV_-mIFNγ glycoproteins in *N. benthamiana* could be achieved. Our study also revealed that TEV protease may efficiently remove the fusion tag from SS^Ext^His(SP)_10_L_TEV_-mIFNγ glycoproteins under biocompatible conditions, releasing C-terminus mIFNγ that underwent self-assembly to biologically active forms with enhanced antivirus activity. Through providing three different cleavable fusion linkers to suit the requirement of different target proteins, the renovated BaMV-based expression system developed in this study offers an alternative strategy for obtaining tag-free therapeutic proteins in compliance with biomedical applications.

## Data Availability

The datasets presented in this study can be found in online repositories. The names of the repository/repositories and accession number(s) can be found in the article/Supplementary Material.
