# Supplementary material for: Development of a tag-free plant-made interferon gamma production system with improved therapeutic efficacy against viruses
Source: Front Bioeng Biotechnol. 2024 Jan 11;11:1341340. doi: 10.3389/fbioe.2023.1341340 (PMC10808299; doi:10.3389/fbioe.2023.1341340)
Supplement: Supplementary file 1 [file DataSheet1.PDF]

Supplementary Material

Supplementary Table 1 | List of primers used in this study.

Primer	Sequence (5'→3')
F-*XbaI*-LPETG mIFNγ	GCTCTAGACTGCCAGAGACTGGT**TGTTACTGCCAGGAC**[2]
F-*XbaI*-ENLYFQG- mIFNγ	GCTCTAGAGAGAACCTTTACTTTCAGGGA**TGTTACTGCCAGGAC**[2]
F-X*baI*-GSHHW mIFNγ	GCTCTAGAGGAAGCCACCACTGG**TGTTACTGCCAGGAC**[2]
R-*SpeI*-TGA-mIFNγ	GCACTAGT**TCACTGGGATGCTCTTCGACC**[2]
F-$(SP)_{10}$-*XbaI*-LPETG	CATCCCCTTCTCCCAGCCCA**TCTAGACTGCCAGAGACTGGT**[2]
F-*MluI*-$(SP)_{10}$	GCACGCGTTCACCCTCTCCAAGCCCTTCCCCATCGCCTAGTCCCTCAC**CATCCCCTTCTCCC**[2]
F-*MluI*-6XHis-GG-(SP)10	GCACGCGTCACCACCACCACCACCACGGTGGA**TCACCC**[2] **TCTCCAAGCCC**[2]
F-*Mlu*I-SS^{Ext}	GCACGCGTATGGGGAAAATGGCTTCTCTATTTGCC**ACTCTTCTAGTAGTTTTAGTGTCA**[2]
R-*Mlu*I-SS^{Ext}	CGACGCGTTGCTGAGCTTTCAGAAGCTAAGCTAAG**TGACACTAAAACTACTAGAAGAGT**[2]

[1] Underlined nucleotides represent the restriction enzyme recognition sites

[2] Nucleotides in bold indicate primer-overlapping regions

Supplementary Figures

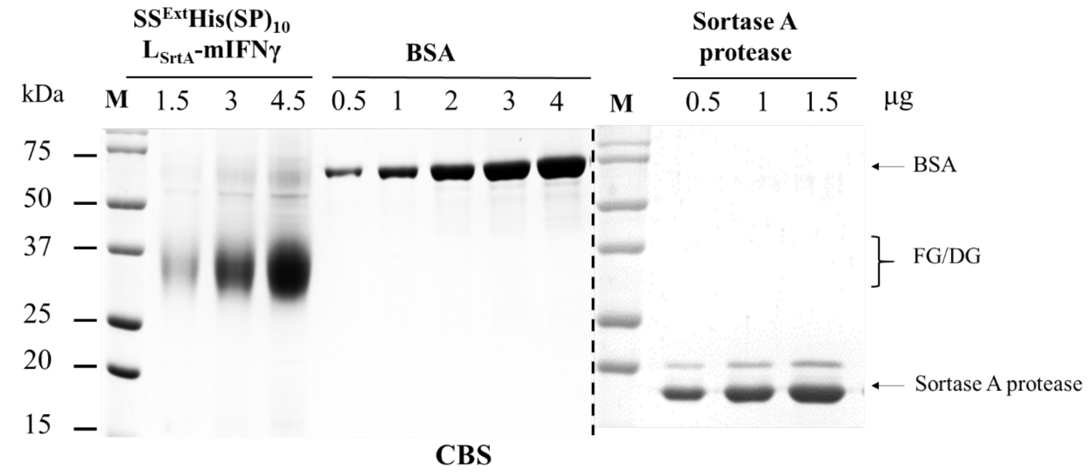

Supplementary Figure 1 | Purification of SS^{Ext}His(SP)$_{10}$L$_{SrtA}$-mIFNγ glycoproteins and Sortase A (SrtA) protease through 1st Ni^{2+}-NTA chromatography. Harvested SS^{Ext}(SP)$_{10}$L$_{SrtA}$-mIFNγ (1.5-4.5 µg) and SrtA (0.5-1.5 µg) fractions after Ni^{2+}-NTA chromatography were analyzed by SDS-PAGE, followed by visualization with CBS. Bovine serum albumin (BSA) proteins (0.5-4 µg) were used as protein concentration standard. M, marker; FG, SS^{Ext}His(SP)$_{10}$L$_{SrtA}$-mIFNγ glycoprotein.

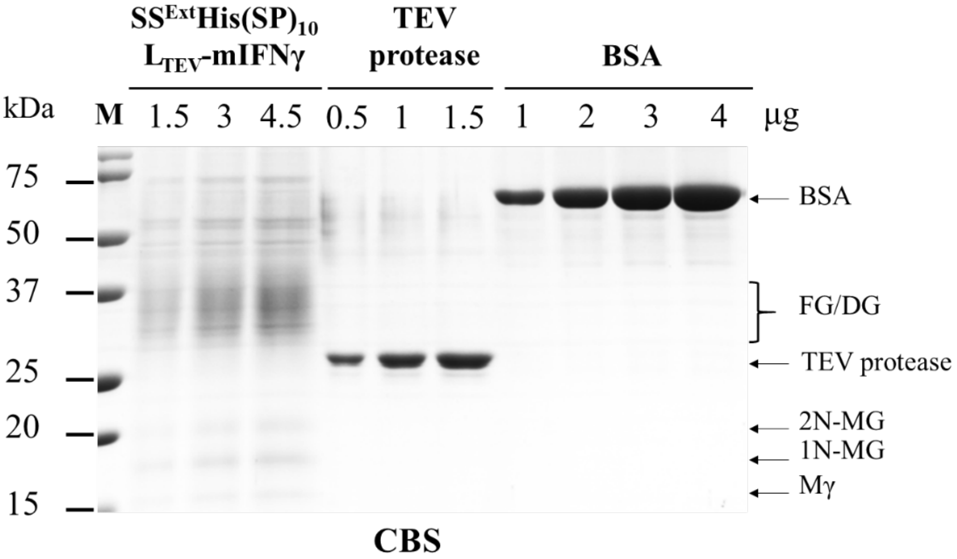

Supplementary Figure 2 | Purification of $SS^{Ext}His(SP)_{10}L_{TEV}$-mIFNγ glycoproteins and tobacco etch virus (TEV) protease through 1st Ni^{2+}-NTA chromatography. Harvested $SS^{Ext}(SP)_{10}L_{SrtA}$-mIFNγ (1.5-4.5 μg) and Sortase A (0.5-1.5 μg) fractions after Ni^{2+}-NTA chromatography were analyzed by SDS-PAGE, followed by visualization with CBS. BSA proteins (1-4 μg) were used as protein concentration standard. M, marker; Mγ , M mIFNγ ; 1N-MG, M monoglycosylated mIFNγ ; 2N-MG, M diglycosylated mIFNγ.

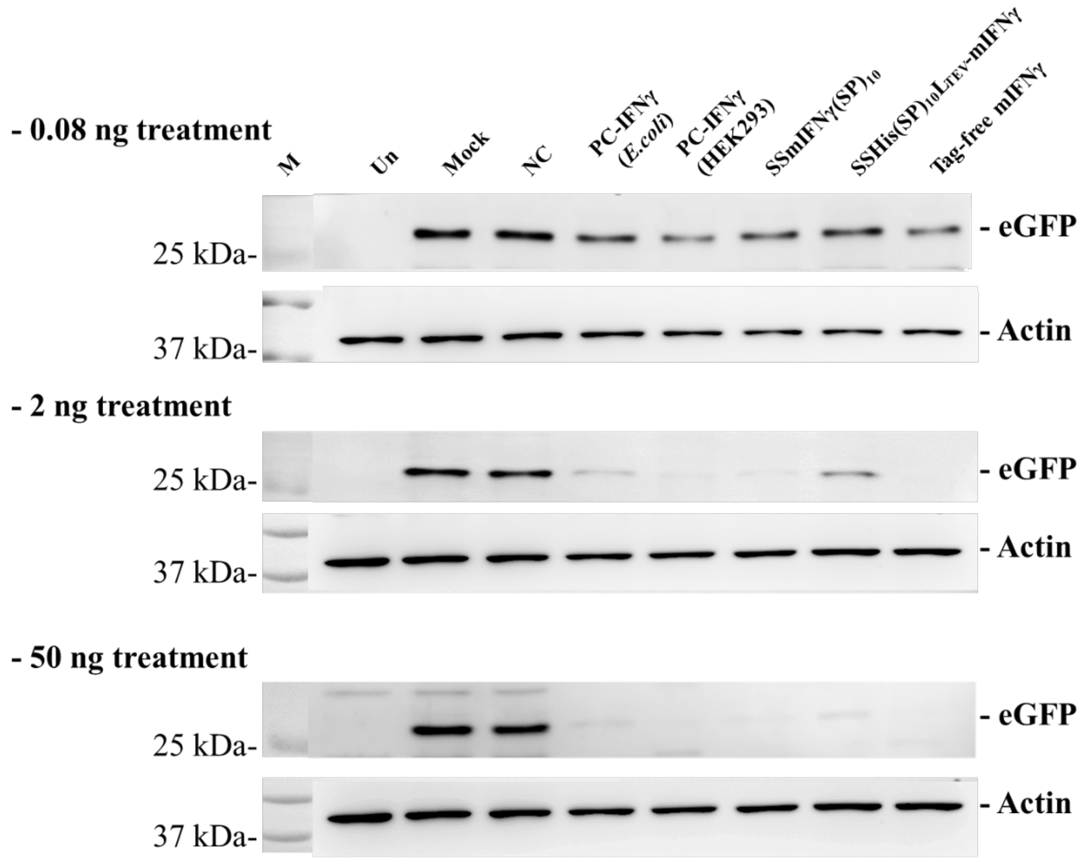

Supplementary Figure 3 | IB analysis of the accumulation of eGFP in anti-viral assay. HEK293-T cells were left uninfected, or pre-treated with DEME (Mock), 50 ng of leaf-extract proteins as the negative control (NC), different concentrations of positive control (PC) proteins, PC-mIFNγ, or PC-IFNγ (commercial IFNγ produced from HEK293), and three tested proteins, SSmIFNγ$(SP)_{10}$, SSHis$(SP)_{10}L_{TEV}$-mIFNγ, or Tag-free mIFNγ, as indicated in the panel, for 12 h. Subsequently, cells were infected with SINV-eGFP at an MOI of 1.0. Infection status was monitored by IB analysis with horseradish peroxidase (HRP)-conjugated eGFP- or actin-specific antibodies at 24 post-infection (hpi).

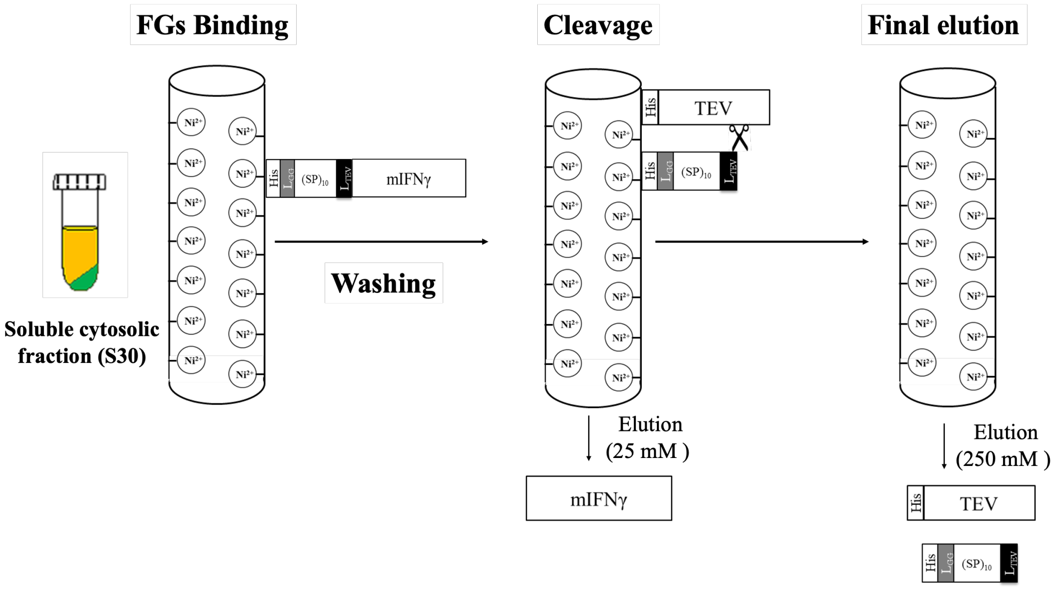

Supplementary Figure 4 | A streamlined purification process of released mIFNγ from $SS^{Ext}His(SP)_{10}L_{TEV}$-mIFNγ glycoproteins. The plant-made fusion proteins are initially bound on the Ni^{2+}-NTA column and separated from the non-target proteins. Following in-column cleavage of the fusion protein with TEV protease, which also contains the 6X His-tag, the tag-free mIFNγ target protein is then eluted (25 mM imidazole) and collected in pure form. In the final elution step (250 mM imidazole), the TEV containing 6X-His-tag may be collected and reused in the subsequent processes.
